# Concise Review: Isoforms of *OCT4* Contribute to the Confusing Diversity in Stem Cell Biology

**DOI:** 10.1002/stem.419

**Published:** 2010-03-23

**Authors:** Xia Wang, Jianwu Dai

**Affiliations:** Key Laboratory of Molecular Developmental Biology, Institute of Genetics and Developmental Biology, Chinese Academy of SciencesBeijing 100190, China

**Keywords:** *OCT4*, OCT4A, OCT4B, OCT4B1, Alternative splicing, Alternative translation initiation

## Abstract

The human *OCT4* gene can generate at least three transcripts (OCT4A, OCT4B, and OCT4B1) and four protein isoforms (OCT4A, OCT4B-190, OCT4B-265, and OCT4B-164) by alternative splicing and alternative translation initiation. OCT4A is a transcription factor responsible for the pluripotency properties of embryonic stem (ES) cells. While OCT4B cannot sustain ES cell self-renewal, it may respond to cell stresses. Yet, the function of OCT4B1 is still unclear. Lack of distinction of *OCT4* isoforms could lead to confusions and controversies on *OCT4* in various tissues and cells. One important issue we emphasize in this review article is that alternatively spliced transcripts and alternative translation products of *OCT4* exhibit diverse expression patterns and functions. Furthermore, simple approaches and methods to detect and distinguish *OCT4* isoforms are discussed. This article underscores the importance of identifying and discriminating the expression and functions of *OCT4* isoforms in stem cell research.

## INTRODUCTION

**Figure 1:**
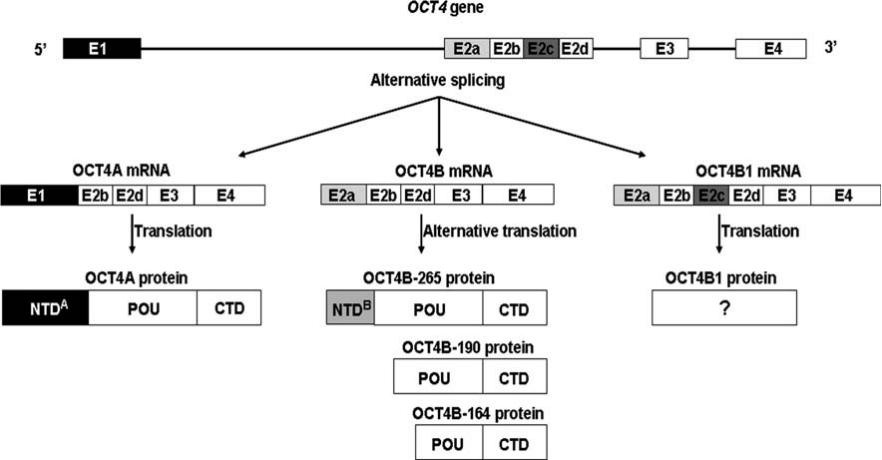
The schematic structure of human *OCT4* gene. *OCT4* gene can generate three transcripts and four protein isoforms. The different regions of *OCT4* isoforms were indicated by different colored boxes, while the identical regions of *OCT4* isoforms were indicated by the same white boxes. Abbreviations: CTD, C-transactivation domain; NTD, N-transactivation domain; POU, a bipartite DNA binding domain.

The pluripotent nature of human embryonic stem (ES) cells provides immense potential to meet many of the clinical demands for regenerative medicine [1,2]. In addition, induced pluripotent stem (iPS) cells promote the progress of clinical application of stem cells [3]. It is well established that *OCT4* gene (official symbol POU5F1, also known as OCT3, OCT3/4, OTF3, and OTF4) functions as a master regulator in maintaining the properties of pluripotency and self-renewal of ES cells [4–6]. *OCT4* is also an essential factor in generating iPS cells [7–9]. The human *OCT4* can generate three main isoforms by alternative splicing, termed OCT4A [10], OCT4B [10] and OCT4B1 [11] (Fig. 1). Since OCT4A and OCT4B variants were identified in 1992 [10], most reports have focused on the study of OCT4A, which has been confirmed as a transcription factor responsible for the stemness properties [4–6,12–17]. However, in recent years, there has been increasing interest in OCT4B, which cannot sustain ES cell self-renewal but may respond to cell stress [12,18–20]. OCT4B1 is a recently discovered *OCT4* spliced variant and it has been considered as a putative marker of stemness [11,21], although the function of OCT4B1 is still unclear.

*OCT4*, which generally refers to OCT4A in most reports, is highly expressed in pluripotent ES cells. The expression of *OCT4* is downregulated during differentiation, and knockdown of OCT4 in ES cells results in differentiation [16,17,22]. Thus, the roles of regulating pluripotency and self-renewal of ES cells endow OCT4 as a pluripotency marker. However, the utility of OCT4 as a marker of pluripotency has been challenged because an increasing number of publications have shown that *OCT4* is expressed in various somatic tissues and cells, such as somatic stem cells, somatic tumor cells, and normal differentiated cells [23–25] (see supporting information Table 1 of Ref.^23^). Several reports argued that detections of *OCT4* in somatic cells are false-positive results due to pseudogene transcripts and DNA contamination [26–29]. In addition, the failure to distinguish *OCT4* isoforms may also lead to the confusions on *OCT4* expression in somatic cells [27,30]. These conflicting results over *OCT4* expression in somatic cells also raise the questions whether *OCT4* functions in maintaining self-renewal of somatic stem cells similarly as that of ES cells and whether it plays a role during oncogenesis.

The importance of discriminating *OCT4* isoforms during the investigation of *OCT4* in various biomedical fields is still not well recognized [31–33]. In this review, we present the argument that alternatively spliced transcripts (OCT4B and OCT4B1) and alternative translation products (OCT4B-190, OCT4B-265, and OCT4B-164) of human *OCT4* may contribute to the expression patterns and functions of OCT4 in various tissues and cells. The possibilities that could cause the confusions of OCT4A by OCT4B are briefly illustrated. In addition, simple approaches and methods used to detect and distinguish the *OCT4* isoforms are discussed. This article underscores the importance of identifying and discriminating the expression patterns and functions of *OCT4* isoforms in stem cell research.

### Isoforms of *OCT4* Generated by Alternative Splicing and Alternative Translation

**Figure 2:**
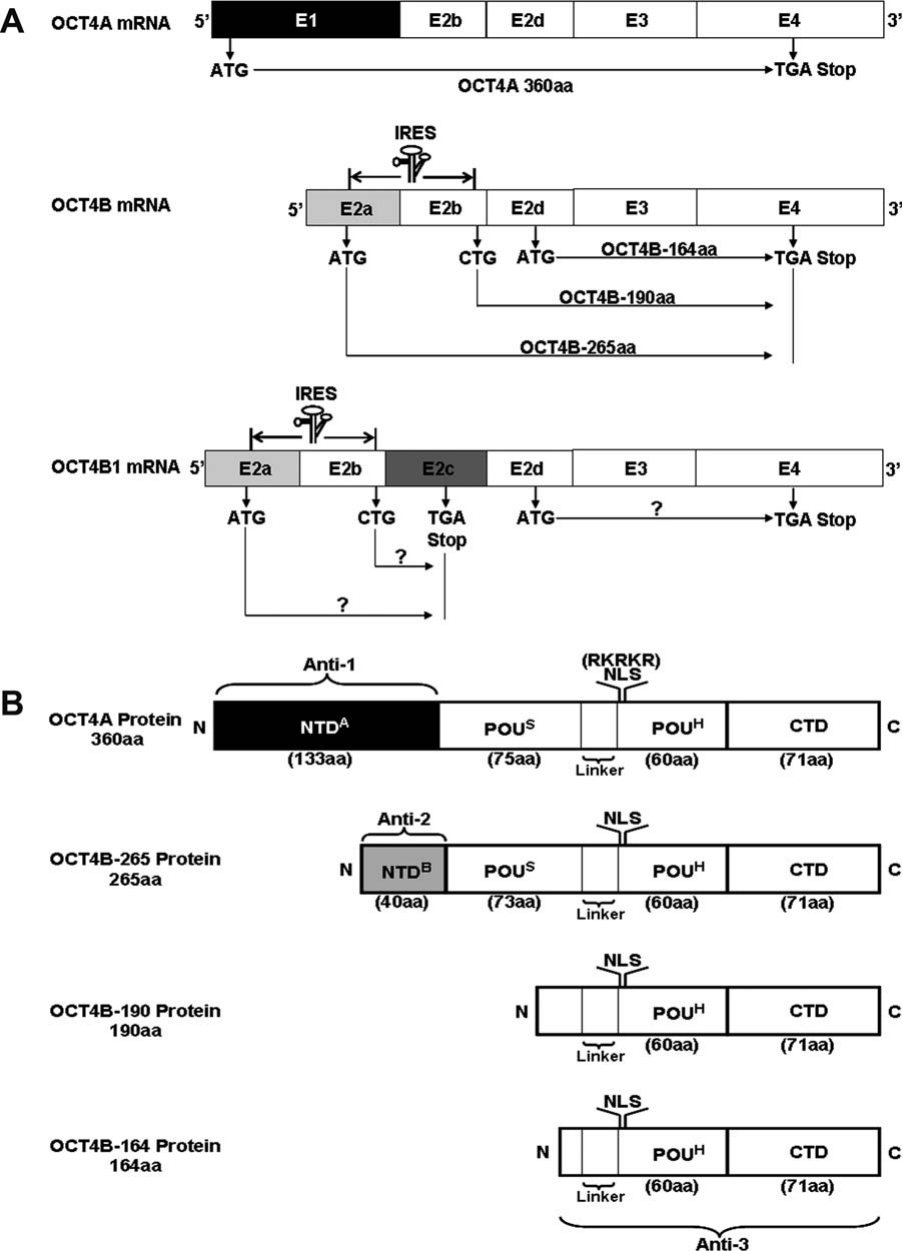
The schematic structure of human *OCT4* transcript and protein isoforms. **(A):** Schematic structure of *OCT4* transcript isoforms. The translation start and stop sites and the putative internal ribosome entry site element on mRNA were indicated, respectively. **(B):** Schematic structure of *OCT4* protein isoforms. The protein domains and the regions recognized by antibodies were showed, respectively. Abbreviations: Anti-1, OCT4A-specific antibody; Anti-2, OCT4B-265-stecific antibody; Anti-3, OCT4 universal antibody; CTD, C-transactivation domain; NTD, N-transactivation domain; IRES, internal ribosome entry site; POU^S^, POU-specific region; POU^H^, POU homeodomain.

**Figure 3:**
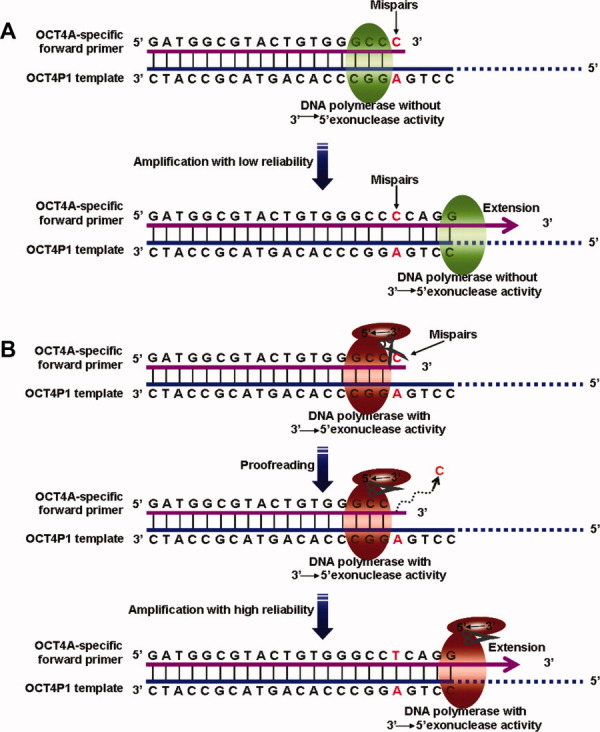
The illustration of the primer with a single-polymorphism at the 3′ end generating mismatched amplification. **(A):** The example of mismatched amplification on OCT4P1 using OCT4A-specific primer by DNA polymerase without 3′ to 5′ exonuclease activity. **(B):** The example of mismatched amplification on OCT4P1 using OCT4A-specific primer by DNA polymerase with 3′ to 5′ exonuclease activity.

The human *OCT4* gene is located on chromosome 6p21.3 [34,35]. Takeda et al. [10] firstly reported that OCT4A (variant 1, NM_002701) and OCT4B (variant 2, NM_203289) were the main variants of *OCT4* gene generated by alternative splicing. OCT4B1 (variant 3, GenBank accession no. EU518650) is the novel variant of *OCT4* gene discovered by Atlasi et al. [11]. As shown by the schematic structures in Figures 1 and 2A, 3 *OCT4* transcript variants are different in 5′ termini and identical in 3′ termini. OCT4A transcript consists of exons 1, 2b, 2d, 3, and 4, among which exon 1 is the unique and special part of OCT4A. In contrast, OCT4B transcript is truncated without exon 1 and specially consists of exon 2a. OCT4B1 transcript is highly identical to OCT4B but consists of an additional exon 2c. *OCT4* gene has been considered to contain five exons. In fact, exons 2a, 2b, 2c, and 2d construct into one entire exon 2 in which several alternative splicing sites are located. Therefore, it may be more accurate to define that *OCT4* gene consists of four exons.

OCT4A is an octamer-binding transcription factor and belongs to the POU family. OCT4A has 360 amino acids and consists of N-transactivation domain (133-amino acids), POU domain (156-amino acids) and C-transactivation domain (71-amino acids) (Fig. 2B). This N-transactivation domain is unique to OCT4A. The POU domain consists of a N-terminal POU-specific region (75-amino acids), a short linker region and a C-terminal homeodomain (60-amino acids) (Fig. 2B) [19,36]. By this bipartite POU DNA-binding domain, OCT4A can specifically bind to the conserved octamer motif (ATTTGCAT) through which OCT4A recognizes the enhancer or promoter regions of its downstream targets [37]. The C-transactivation domain is a cell-type-specific domain and its transactivation activity is mediated by the POU domain [38].

It has been thought that the protein product of OCT4B is composed of 265 amino acids [10,12,19,27]. And it is thought that certain population cannot express OCT4B protein because of a single nucleotide polymorphism site in the OCT4B start codon (A*T*G→A*G*G) [10]. However, Wang et al. [20] recently identified a putative internal ribosome entry site element present in exon 2 a–b region of OCT4B mRNA, and OCT4B transcript can be translated starting from the internal site of the mRNA. Thus, by alternative translation initiation, a single OCT4B transcript may encode at least three protein isoforms: OCT4B-265 (initiation from the first ATG start codon and consisting of 265-amino acids), OCT4B-190 (initiation from the CTG start codon and consisting of 190-amino acids), and OCT4B-164 (initiation from the second ATG start codon and consisting of 164-amino acids) (Fig. 2A). OCT4B-265 protein isoform contains a unique N-transactivation domain (40-amino acids) which is different from that of OCT4A (Fig. 2B). The POU domain of OCT4B-265 is 2-amino acid shorter than that of OCT4A and its C-transactivation domain is identical to OCT4A. OCT4B-190 protein isoform consists of a truncated POU domain (119-amino acids), while the POU homeodomain (60-amino acids) and C-transactivation domain (71-amino acids) remain intact and are completely identical to those of OCT4A and OCT4B-265 (Fig. 2B). OCT4B-164 is the shortest protein isoform but it still has full POU homeodomain and C-transactivation domain (Fig. 2B).

The protein product of OCT4B1 has not been identified yet. An in-frame stop codon TGA is located in the additional exon 2c of OCT4B1 which is spliced out in the OCT4B mRNA (Fig. 2A). As a result, OCT4B1 transcript cannot encode the full length of OCT4B-265 and OCT4B-190 protein isoforms, although it remains unclear whether OCT4B1 transcript can yield the truncated peptides of these 2 isoforms.

In addition, there may be some other undiscovered *OCT4* isoforms. Liedtke et al. [26] have obtained 13 mRNA by examining the UniGene cluster for *OCT4* (NM002701) and using BLASTn search for single exons of *OCT4*. Among the 13 *OCT4* transcripts, OCT4A (NM002701) and OCT4B (NM203289) are listed, whereas OCT4B1 is not involved. Interestingly, OCT4B1 is likely to be a truncated product of the transcript DQ486514 [26]. Thus, considerable efforts are needed to be devoted to identifying and detecting novel *OCT4* spliced variants and other *OCT4*-related genes in the future.

### Methods Used to Detect and Distinguish *OCT4* Isoforms

To elucidate the expression patterns and the biological functions of *OCT4* gene, the principal and important task is to detect and discriminate each of *OCT4* isoforms. Our analysis will be focused on the three known isoforms of *OCT4*: OCT4A, OCT4B, and OCT4B1.

RT-PCR and quantitative real-time RT-PCR are usually used to detect *OCT4* expression at mRNA levels. Liedtke et al. [26,27] and de Jong et al. [28] have emphasized that nonspecific primers can result in false-positive artifacts and misinterpretations in stem cell research, unfortunately the lack of significant considerations of this problem still exists in many studies. Several important approaches on the detection of OCT4A transcript are summarized as follows:

a. To discriminate from other currently known splice variants of *OCT4*: Since exon 1 is unique in OCT4A transcript, the forward primer used to detect OCT4A should lie in exon 1 to distinguish OCT4A from OCT4B and OCT4B1 variants, and the primers should be intron spanning to avoid PCR amplification from *OCT4* genome sequence.

b. To avoid DNA contamination and confusion from pseudogenes: The six currently known pseudogenes which are highly homologous to *OCT4* cause many problems in detecting the expression of OCT4A transcript [26,39]. The processed pseudogenes lack introns, which arise through mRNA retrotransposition into the genome [40]. Therefore, the RNAs should be completely treated with RNase-free DNaseI to remove DNA contamination. Panagopoulos et al. [41] have evoked the notion that the RNA, even after DNAse treatment, may be not completely free of DNA, and trace amount of DNA might cause artifacts in subsequent PCR amplifications. Thus, RT-PCR should be carried out without reverse transcriptase as a negative control. Furthermore, some *OCT4* pseudogenes are transcribed in vivo [42], and these transcribed pseudogenes can be mainly responsible for RT-PCR artifacts. Accordingly, it seems that the best approach is to design specific primers for OCT4A transcript in exon 1, excluding all pseudogenes of *OCT4.*

c. To design specific primers for OCT4A: Liedtke et al. [26] recommended two pairs of primers that may exclude amplification of all unwanted transcripts, and some publications have utilized these primers to examine the expression of OCT4A [43,44]. One forward primer (Oct4_F_P 5′-GATGGCGTACTGTGGGCC*C*-3′) carries a polymorphism at the 3′ end to discriminate OCT4A from the other varians and the pseudogenes on chromosomes 1 and 8. Nevertheless, it should be cautious when using a primer to distinguish sequences with a single nucleotide variance. Because *Taq* DNA polymerase, being devoid of 3′ to 5′ exonuclease activity, can make nucleotide extension from the mismatch at the 3′ end of primer by the low reliability [45,46] (Fig. 3A). In addition, polymerases with 3′ to 5′ exonuclease activity, such as Pfx or Pfu DNA polymerase, can proofread the mispairs on the 3′ end of primer by removing the mismatched nucleotide and subsequently make extension along the template [46] (Fig. 3B). Therefore, it is important to note that this OCT4A-specific primer [26], making use of a single polymorphism at the 3′ end, can still mismatch to the pseudogenes under certain conditions, especially when the amount of OCT4A transcripts is low. Some researchers have designed the “OCT4A-specific” forward primer at the beginning of 5′ end of the OCT4A mRNA which had been considered as a region absent in all of the *OCT4* pseudogenes [20,26] (supporting information Fig. S1). But, we recently found that the novel published mRNA sequence of *OCT4* pseudogene OCT4P1 (official symbol POU5F1B, NM_001159542) is largely extended at the 5′ end compared to the older sequence (NR002304) (supporting information Fig. S1). As a result, those forementioned “OCT4A-specific” forward primers may also recognize OCT4P1 and lose their specificities to OCT4A. Thus, it may be indispensable to design a specific forward primer for OCT4P1 in the special 5′ end of OCT4P1 mRNA as a control during the RT-PCR exam of OCT4A transcript (supporting information Fig. S1). It is also necessary to verify the PCR products by sequence analysis, as there are some nucleotide mutations in pseudogenes different from the parental gene OCT4A (supporting information Fig. S1). Furthermore, in each experiment, a positive control (human ES cells or embryonic carcinoma cell lines, etc.) and a negative control (human adult fibroblast cells, etc.) should be used at the same time.

d. Alternative approaches for the detection of OCT4A: OCT4A contains *ApaI* and *Tsp45I* restriction sites in exon 1 and these two sites are unique to OCT4A and are not present in the currently known pseudogenes or other splice variants. Panagopoulos et al. [41] have proposed a PCR/restriction digestion assay to identify OCT4A specially. More optimal strategies and methods for detection of OCT4A transcript should be explored in the future.

Detection of OCT4B and OCT4B1 isoforms at mRNA level is much easier than that of OCT4A, as no pseudogenes for OCT4B and OCT4B1 isoforms have been identified in the genome currently [28]. Exon 2a is absent in OCT4A and primers located in this region can discriminate OCT4B and OCT4B1 from OCT4A. Exon 2c is unique to OCT4B1 and OCT4B1-specific primers can be designed in this region. Additionally, when forward primer and reverse primer span exon 2c, we can distinguish between OCT4B and OCT4B1 transcripts by the different size of PCR products. For the detection of OCT4B and OCT4B1, it is necessary to design intron-spanning primers, to remove DNA contamination and to verify PCR products by sequence analysis.

Finally, for all of the quantitative real-time RT-PCR, the dissociation curve should be used to control the specificity of PCR reaction. With regard to in situ hybridization, RNA interference, microarray and gene knockout, etc., the probe sequence or target sequence should be clearly defined to each *OCT4* isoforms.

Since mRNA expression at a comparatively basal level does not account for the functional protein expression, it is necessary to confirm *OCT4* expression at protein level. However, many publications did not examine the *OCT4* protein expression [23] (see supporting information Table 1 of Ref.^23^). It should be noted that improper antibody and methods could also lead to misleading results on *OCT4*. *OCT4* protein isoforms are identical in C-terminal region and distinct in N-terminal region, so the location of the epitope recognized by the anti-OCT4 antibody should be considered at first. Methods used most frequently to detect *OCT4* protein are immunohistochemistry and immunocytochemistry. By these methods, we can specifically identify OCT4A and OCT4B-265 using antibodies mapping at the N-terminal of OCT4A or OCT4B-265, respectively. However, OCT4B-190 and OCT4B-164 do not have special parts in their protein sequence, so they cannot be specifically separated from OCT4A and OCT4B-265 by these methods. Therefore, it is necessary to identify each isoforms through the different sizes of *OCT4* protein bands by Western blot analysis. It should be taken into account that nonspecific proteins might lead to false-positive results, in turn positive control and negative control are both indispensable, and monoclonal antibodies are recommended.

Because of the protein product of OCT4P1, which is composed of 359 amino acids and with 95% homology to OCT4A [47], the detection of OCT4A protein expression becomes more problematic. To discriminate between OCT4A and OCT4P1, it might be helpful to base on the RT-PCR results by using OCT4P1-specific primer or a PCR/restriction digestion assay as mentioned above, and then to determine the methylation status of the promoter and enhancer regions of *OCT4* and *OCT4P1*, respectively. An antibody which can directly distinguish between OCT4A and OCT4P1 will be desirable in the future.

### The Cellular Localization of OCT4 Isoforms

It is well known that OCT4A protein is localized in the nucleus as a transcription factor. Cauffman et al. [19] and Lee et al. [12] both showed that OCT4B protein was located in the cytoplasm. Therefore, some researchers may believe that OCT4A and OCT4B can be distinguished by their distinct localization. However, Wang et al. [20] found that OCT4B-265, OCT4B-190, and OCT4B-164 proteins were all diffusely localized in both cytoplasm and nucleus. These inconsistent results may reflect the complicated regulation of *OCT4*, as most alternative initiation of translation generates functional diversity through regulating the localization of protein isoforms [48–50]. Noticeably, a putative nuclear localization signal (RKRKR) [51] remains in all protein isoforms of *OCT4*, and the localization change of OCT4B-190 is closely correlated with the function of OCT4B-190 under stress [20]. Consequently, the localization of *OCT4* protein isoforms may be closely regulated in accordance with their diverse functions.

### *OCT4* Isoforms in Pluripotent Stem Cells During Human Embryogenesis

As we all know, *OCT4* is vital for the formation of pluripotent stem cells in inner cell mass and plays a critical role in mammalian early embryonic development [4–6,13,14,52]. Nearly all of the reports referring *OCT4* as OCT4A, until 2005, Cauffman et al. [18] investigated the expression pattern of *OCT4* throughout the human preimplantation development, considering both OCT4A and OCT4B for the first time. In blastocysts, OCT4A and OCT4B showed different spatial expression patterns within a cell. Based on these findings, Cauffman et al. [18] suggested that *OCT4* protein isoforms may show different functional properties. Subsequently, Cauffman et al. [19] further discriminated the expression patterns between OCT4A and OCT4B in human ES cells and all stages of preimplantation development by immunocytochemistry. OCT4A was expressed in all nuclei of compacted embryos and blastocysts, whereas OCT4B was expressed in the cytoplasm of all cells from the four-cell stage onwards. In this report, Cauffman et al. speculated that the stemness properties of *OCT4* could be ascribed to OCT4A but not to OCT4B. This statement was subsequently supported by Lee et al. [12]. They showed that only OCT4A isoform was responsible for the stemness properties, whereas OCT4B was not sufficient to sustain ES cell self-renewal or to maintain ES cell undifferentiated state. OCT4B isoform did not bind to the octamer motif of OCT4A because there are two regions within the N-transactivation domain of OCT4B inhibiting DNA binding. Furthermore, OCT4B could not activate transcription of the downstream genes regulated by OCT4A [12]. It is still unclear whether OCT4B-190, losing the N-transactivation domain and holding a truncated POU domain, has the DNA binding ability.

In human ES cells, transcripts of the three main isoforms of *OCT4* were all detected. OCT4A mRNA was eightfold more abundant than OCT4B [12], while OCT4B1, like OCT4A, was highly expressed in human ES cells and was downregulated following differentiation [11]. At protein level, OCT4B-190 protein was recently found to be upregulated in human ES cells under heat shock by Western blot analysis. No expression of OCT4B-265 and OCT4B-164 protein isoforms was detected in human ES cells [20]. The expression patterns and functional properties of OCT4B, referring to those three protein isoforms, should be re-estimated in human early embryonic development.

### *OCT4* Isoforms in Primodial Germ Cells and Germ Cell Tumors

Human primordial germ cells (PGCs) can be a source of pluripotent stem cells, such as embryonic germ cells. *OCT4* is also highly expressed in PGCs [53–56], but many studies have not distinguished *OCT4* isoforms [53–56]. Studies in mouse have demonstrated that *Oct4* is required for PGCs survival in vivo [57]. These data suggest that the *OCT4* isoforms may play vital roles in PGCs.

Certain types of germ cell tumors (GCTs) have pluripotent potential, such as embryonal carcinoma (EC), seminoma, and carcinoma in situ. *OCT4* is present in these “pluripotent” GCTs but absent in the other differentiated GCTs such as teratoma and yolk sac tumor [28,58–64]. *OCT4* shows strong nuclear immunostaining and it has become the most sensitive and informative biomarker in the diagnosis of those “pluripotent” GCTs [28,60,62,63,65]. However, the vast majority of publications on *OCT4* expression in GCTs have not specified *OCT4* isoforms. For example, many studies detected the expression of *OCT4* by immunohistochemistry using the polyclonal antibody (sc-8629; Santa Cruz Biotechnology Inc., Santa Cruz, CA, http://www.scbt.com) which can recognize all four currently known protein isoforms of *OCT4* [58–61,63]. It is important to note that weak cytoplasm staining for OCT4 was clearly seen in many reports using this polyclonal antibody (sc-8629; Santa Cruz Biotechnology Inc.) [58,61–63], and cytoplasmic staining has been considered as an intrinsic character of EC [62]. These raise the possibility that other *OCT4* protein isoforms might be also present in GCTs, besides OCT4A. Recent studies on human EC cell lines showed that OCT4B transcript was expressed at a low level [20] and OCT4B1 transcript was expressed at a higher level [11]. OCT4B-190 protein was upregulated under stress in EC cells although the amount of OCT4B-190 protein was very low [20].

### *OCT4* Isoforms in Somatic Stem Cells

There have been significant efforts in searching for markers of somatic stem cells, *OCT4*, being the well-known pluripotency marker, has been widely explored in somatic stem cells [23] (supporting information Table 1 of Ref.^23^). Although considerable studies have showed positive results for *OCT4* expression in a variety of somatic stem cells, most studies did not exclude the possibilities of pseudogene artifacts and did not distinguish *OCT4* isoforms [27,31] (see examples in table 1 of Ref.^27^).

Recently, Mueller et al. [66] investigated *OCT4* expression in human mesenchymal stem cells (MSC) by RT-PCR and Western blot analysis. No reliable expression of OCT4A was obtained using two pairs of primers and two types of antibodies specific for OCT4A, however positive results were found using primers for all the three isoforms of *OCT4*. These data provide the possibility for the expression of OCT4B or OCT4B1 in MSC. Furthermore, Kaltz et al. [67] have provided convincing evidence for *OCT4* expression in human bone marrow-derived MSC (BM-MSC) and umbilical vein-derived stromal cells (UVSC). They claimed that *OCT4* transcripts can indeed be detected by PCR in BM-MSC and UVSC, while almost all of the *OCT4* transcripts corresponded to OCT4B (OCT4A/OCT4B ratio, 0.2 in BM-MSC and 0.18 in UVSC). OCT4A PCR products were verified by cloning and sequencing analyses, and data showed that more than 89% of OCT4A transcripts corresponded to pseudogene OCT4P1, OCT4P3, or OCT4P4. Combined with the real-time results, OCT4A was expressed in BM-MSC and UVSC at a level of 8,730-fold and 4,305-fold lower than that in EC cells, respectively. Although basal level of OCT4A transcripts were detected in BM-MSC and UVSC, immunoprecipitation and Western blot analyses failed to show any band corresponding to OCT4A protein by using the OCT4A-specific antibody. The putative proteins of pseudogene OCT4P3 and OCT4P4 were not detected by the same antibody [67]. So far, proteins of OCT4B and OCT4B1 have not been explored yet in somatic stem cells, and it remains a question whether OCT4B and OCT4B1 function in various types of somatic stem cells.

Greco et al. [68] reported that *OCT4* functioned through similar regulatory pathways in human MSCs and ES cells, but they did not discriminate the isoforms of *OCT4*, nor did they exclude the pseudogenes of *OCT4*. One unexpected result in this report was that the protein amount of OCT4A in MSCs was higher than that in human ES cells. Lengner et al. [23] provided considerable evidence for *Oct4* expression in somatic stem cells in mouse. They determined that *Oct4* was not responsible for self-renewal or maintaining pluripotency in somatic stem cells deriving from various tissues including the intestinal epithelium, BM (mesenchymal and hematopoietic stem cells), hair follicle, brain, and liver. These results suggested that “stemness” marker Oct4A may not be applicable to somatic stem cells. It is important to note that exon 1 of Oct4 was deleted by the tissue-specific recombination resulting in Oct4A inactivation, while the expression of other isoforms of Oct4 in mouse may not be affected. Since several novel isoforms of *Oct4* have recently been identified in mouse somatic cells [69], further studies need to be performed for the functions of *Oct4* isoforms.

### *OCT4* Isoforms in Human Somatic Tumors and Tumor Cells

OCT4 may play critical roles in the oncogenesis of human GCTs [64], and ectopic expression of Oct4 may cause dysplasia in epithelial tissues in mouse [70]. In addition, under the hypothesis of “cancer stem cell” in somatic tumors, an increasing number of researchers explored the expression of *OCT4* in human somatic tumors and somatic tumor cell lines [23,25,32,33,44,71] (supporting information Table 1 of Ref.^23^). Failure to discriminate these isoforms of *OCT4* also leads to confusing results in cancer cells [25,32,33,72–74]. Cantz et al. [75] have confirmed that OCT4A could not be detected in the nucleus of Hela and MCF7 cells by immunofluorescence and Western blot analyses using two different monoclonal antibodies. Compared to that of EC cells, OCT4A transcripts could not be so significantly detected in Hela and MCF7 cells by RT-PCR. Additionally, the distal enhancer region of OCT4 promoter was highly methylated in Hela, MCF7, HePG2, and OS732 cancer cell lines, compared with EC cell lines [20,75]. Furthermore, Mueller et al. [66] analyzed OCT4 expression in 42 human somatic tumor cell lines by RT-PCR, Western blotting, immunocytochemistry and immunohistochemistry using three pairs of primers and three different antibodies. They demonstrated that OCT4A protein was not present in any of the 42 somatic tumor cell lines; in contrast, OCT4B or other splice variants were likely expressed in these somatic tumor cells. Wang et al. [20] recently provided evidence that OCT4B-190 might protect Hela cells against apoptosis under stress. More investigations are required to further characterize the expression patterns and functions of OCT4B protein isoforms in human somatic tumors and cell lines.

When studying *OCT4* expression in somatic tumors and tumor cells, OCT4P1 should be taken into account, because OCT4P1 (POU5F1P1) may be associated with increased risk for prostate cancer [76]. Monsef et al. [71] demonstrated that cytoplasmic OCT4B isoform was present in prostate cancer and benign prostate hyperplasia. Neither transcripts nor nuclear protein of OCT4A was detected by RT-PCR/restriction digestion assay, immunohistochemistry and Western blot analysis with two different antibodies. In contrast, Sotomayor et al. [44] reported that OCT4A was expressed in prostate cancer. The conflicting results might be due to false-positive results caused by OCT4P1, as OCT4P1 may not be excluded in the latter study [44].

### *OCT4* Isoforms in Normal Differentiated Cells

In 2007, Zangrossi et al. [24] challenged the role of OCT4 as a real pluripotency marker, as they showed that OCT4 expression in human peripheral blood mononuclear cells which are terminally differentiated cells. However, Kotoula et al. [30] revised the experimental data of Zangrossi et al. and presented the reasons that OCT4B variant, but not OCT4A variant, might be expressed in peripheral blood mononuclear cells. Subsequently, Panagopoulos et al. [41] supported this result by demonstrating that only OCT4B variant was expressed in 10 samples of peripheral blood leukocytes.

### *OCT4* Isoforms in Reprogramming and iPS Cells

**Figure 4:**
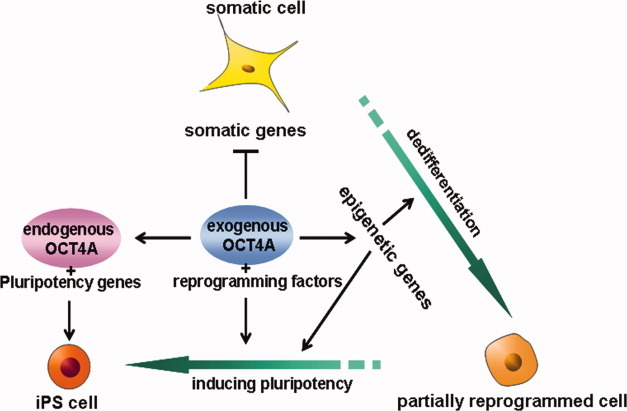
The schematic illustration for the role of OCT4A in reprogramming of somatic cells. Exogenous OCT4A and the other reprogramming factors may directly and indirectly silence somatic genes and reactivate pluripotency genes during reprogramming process. Endogenous OCT4A and the other pluripotency genes can maintain the pluripotent state of induced pluripotent stem cells. Abbreviation: iPS, induced pluripotent stem (cells).

It has been established that OCT4A, in close collaboration with SOX2 and NANOG, govern the transcriptional regulatory network in maintenance of ES cell pluripotency by activating expression of pluripotency-related genes and repressing expression of differentiation-related genes [6]. OCT4A also appears to be the most important determinant at the transcriptional level during reprogramming of human somatic cells into iPS cells [7,8,77] (Fig. 4).

Exogenous OCT4A, together with other reprogramming factors, may directly and indirectly silence somatic lineage-associated genes and reactivate ES cell-specific genes in somatic cells. Oct4 is associated with polycomb complexes which can remodel chromatin to obstruct the transcription of genes involved in lineage commitment [78,79], thus exogenous OCT4A may induce the expression of repressive epigenetic genes to repress lineage-associated genes in somatic cells during reprogramming. Oct4 may also function in X-chromosome reprogramming, which is necessary for the dedifferentiation of somatic cells to iPS cells [80]. Furthermore, it has been shown that chromatin remodeling and histone-modifying complexes (e.g., SMARCAD1, MYST3, and SET) are co-occupied by OCT4, SOX2, and NANOG [6]. Thus, it is conceivable that exogenous OCT4A may contribute to the genome-wide epigenetic modifications for derivation of iPS cells. Since the H3K9Me2 and H3K9Me3 demethylase genes are directly regulated by Oct4 in ES cells [81], it is possible that exogenous OCT4A also plays a vital role in DNA demethylation which is essential for the reactivation of pluripotency genes during reprogramming. It has been demonstrated that the co-binding of Oct4, Sox2, and Klf4 can activate pluripotency genes during reprogramming [82]. Interestingly, both the silencing of exogenous OCT4 and the reactivation of endogenous OCT4 are essential for generating the fully reprogrammed iPS cells [83,84]. Finally, endogenous OCT4A, together with endogenous SOX2 and NANOG, re-establish the autoregulatory loop and transcriptional network to maintain the pluripotent state of iPS cells.

Currently, there is no evidence available that OCT4B and OCT4B1 isoforms are involved in the generation of iPS cells. Since OCT4B-190 is upregulated in human ES cells under stress and may play a role in protection against apoptosis [20], one could speculate that OCT4B-190 may participate in the reprogramming process as an antiapoptosis factor.

In addition, the precise expression level of Oct4 has been shown to be critical for maintaining a self-renewing pluripotent state in ES cells [5], and the efficient reprogramming of somatic cells is also highly sensitive to the precise relative amount of OCT4 [85]. It is interesting to know if OCT4B and OCT4B1 effect on the OCT4A expression at mRNA and protein levels.

## CONCLUSION

Human somatic stem cells, somatic tumor cells, and some adult cells may indeed express OCT4A mRNA at a basal level, compared with pluripotent cells. Nevertheless, the functional protein of OCT4A has not been reliably detected in the nonpluripotent cells. It is not clear whether the basal-level expression of OCT4A still be endowed other biological functions in nonpluripotent cells. However, the high-level expression of OCT4A protein still remains a property for pluripotent cells.

OCT4B is expressed at low levels in human somatic stem cells, tumor cells, adult tissues, as well as pluripotent cells. It is possible that OCT4B has diverse functions in different cells by various protein products. OCT4B is likely to play a role under stress response [20], and more detailed biological characterization of OCT4B should be explored in the future. It is interesting to note that OCT4B1 may be related to stemness [11,21], and investigations for OCT4B1 would contribute to stem cell research.

Each isoform of *OCT4* reveals distinct sequence, thus OCT4A, OCT4B, and OCT4B1 may contain different RNA regulatory elements. Possibly, *OCT4* isoforms may use different promoter and enhancer elements for their transcription. Therefore, *OCT4* isoforms could be regulated respectively under the same conditions. However, it is possible that protein isoforms of *OCT4* may have close connections with each other in biological functions due to the common sequence they shared. It is important to note that OCT4 isoforms are simultaneously expressed in some cells (e.g., human ES cells), hence it is conceivable that OCT4 isoforms may interact each other by competitive or synergistic interaction at transcriptional and/or post-transcriptional levels. The possibility and mechanisms remain unexplored.

**Figure 5:**
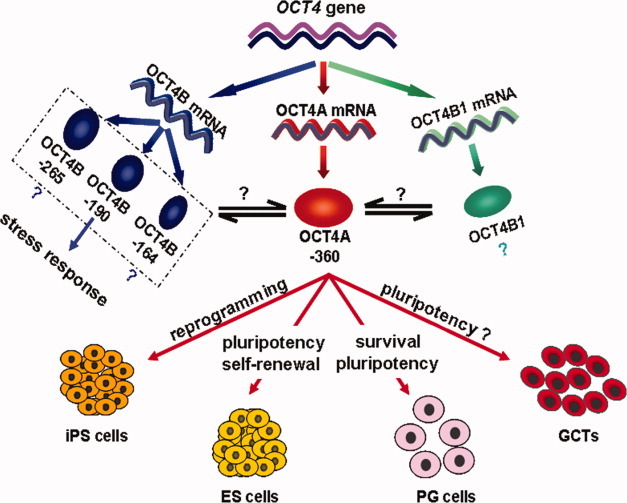
The schematic illustration of the relation of OCT4 isoforms to variant cells. Isoforms of OCT4 show the diversity in different cells. Abbreviations: iPS, induced pluripotent stem (cells); ES, embryonic stem (cells); PG, primordial germ (cells); GCTs, germ cell tumors.

The results summarized in this review suggest that *OCT4* isoforms, not only OCT4A but also OCT4B and OCT4B1, contribute to the expression patterns and functions of OCT4 gene in a variety of human cells (Fig. 5). Like other crucial genes (such as *FGF-2*, *VEGF, C-myc*), *OCT4* can generate transcript variants by alternative splicing and protein diversity by alternative translation initiation to overcome the limited number of genes in the genome and to perform multiple biological functions. To better understand OCT4, it is crucial to identify and distinguish its isoforms of *OCT4* in expression patterns as well as function varieties in stem cell biology.
