# Supplementary material for: Concise Review: Isoforms of OCT4 Contribute to the Confusing Diversity in Stem Cell Biology
Source: Stem Cells. 2010 Mar 23;28(5):885–93. doi: 10.1002/stem.419 (PMC2962909; doi:10.1002/stem.419)
Supplement: Supplementary file 1 [file stem0028-0885-SD1.doc]

**Supplementary Figure 1**

Sequence alignment of human OCT4A mRNA (NM_002701) and OCT4P1 mRNA (NM_001159542). Boxed sequence showed the previous OCT4A-specific forward primer (5'-TCCCTTCGCAAGCCCTCATT-3') used by Wang et al.^1^, and shadowed sequence showed the other forward primer (Oct4_F 5'-AGCCCTCATTTCACCAGGCC-3') used in Liedtke et al.^2^. The blue sequence is unique to OCT4P1 and specific primers for OCT4P1 can be designed in this region. The *** indicated the start site of OCT4P1 older sequence (NR002304).

OCT4A (1) --------------------------------------------------

OCT4P1 (1) AACATTTCCAAATCTTGGCATTCTTATCCACAAAGTGAAGATAATAATTG

OCT4A (1) --------------------------------------------------

OCT4P1 (51) TCAATTCACAGGTGATTATGATTTAAAGAGATTACTTTTGAAGAGTTCCT

OCT4A (1) --------------------------------------------------

OCT4P1 (101) AACACATTCAGTCAACATTTAATGATGCTTCAGGCACTGTGTTCATTGCT

OCT4A (1) --------------------------------------------------

OCT4P1 (151) AGTGAGCGTATGACACACACAGCCATACGGTCACAGAGCTTTCAATGAAA

OCT4A (1) -CCTTCGCAAGCCCTCATTTCACCAGGCCCCCGGCTTGGGGCGCCTTCCT

OCT4P1 (201) AGTAACATAATTGCTCATTTCACCAGGCCCCCGGCTTGGGGCGCCTTCCT

OCT4A (50) TCCCCATGGCGGGACACCTGGCTTCGGATTTCGCCTTCTCGCCCCCTCCA

OCT4P1 (251) TCCCCATGGCGGGACACCTGGCTTCGGATTTCGCCTTCTCGCCCCCTCCA

*******

OCT4A (100) GGTGGTGGAGGTGATGGGCCAGGGGGGCCGGAGCCGGGCTGGGTTGATCC

OCT4P1 (301) GGCGGTGGGGGTGATGGGCCATGGGGGGCGGAGCCGGGCTGGGTTGATCC

OCT4A (150) TCGGACCTGGCTAAGCTTCCAAGGCCCTCCTGGAGGGCCAGGAATCGGGC

OCT4P1 (351) TCTGACCTGGCTAAGCTTCCAAGGCCCTCCTGGAGGGCCAGGAATCGGGC

OCT4A (200) CGGGGGTTGGGCCAGGCTCTGAGGTGTGGGGGATTCCCCCATGCCCCCCG

OCT4P1 (401) CGGGGGTTGGGCCAGGCTCTGAGGTGTGGGGGATTCCCCCTTGCCCCCCG

OCT4A (250) CCGTATGAGTTCTGTGGGGGGATGGCGTACTGTGGGCCCCAGGTTGGAGT

OCT4P1 (451) CCGTATGAGTTATGTGGGGGGATGGCGTACTGTGGGCCTCAGGTTGGAGT

OCT4A (300) GGGGCTAGTGCCCCAAGGCGGCTTGGAGACCTCTCAGCCTGAGGGCGAAG

OCT4P1 (501) GGGGCTAGTGCCCCAAGGCGGCTTGGAGACCTCTCAGCCTGAGAGCGAAG

OCT4A (350) CAGGAGTCGGGGTGGAGAGCAACTCCGATGGGGCCTCCCCGGAGCCCTGC

OCT4P1 (551) CAGGAGTCGGGGTGGAGAGCAACTCCAATGGGGCCTCCCCGGAACCCTGC

OCT4A (400) ACCGTCACCCCTGGTGCCGTGAAGCTGGAGAAGGAGAAGCTGGAGCAAAA

OCT4P1 (601) ACCGTCCCCCCTGGTGCCGTGAAGCTGGAGAAGGAGAAGCTAGAGCAAAA

OCT4A (450) CCCGGAGGAGTCCCAGGACATCAAAGCTCTGCAGAAAGAACTCGAGCAAT

OCT4P1 (651) CCCGGAGAAGTCCCAGGACATCAAAGCTCTGCAGAAAGAACTCGAGCAAT

OCT4A (500) TTGCCAAGCTCCTGAAGCAGAAGAGGATCACCCTGGGATATACACAGGCC

OCT4P1 (701) TTGCCAAGCTCCTGAAGCAGAAGAGGATCACCCTGGGATATACACAGGCC

OCT4A (550) GATGTGGGGCTCACCCTGGGGGTTCTATTTGGGAAGGTATTCAGCCAAAC

OCT4P1 (751) GATGTGGGGCTCATCCTGGGGGTTCTATTTGGGAAGGTGTTCAGCCAAAA

OCT4A (600) GACCATCTGCCGCTTTGAGGCTCTGCAGCTTAGCTTCAAGAACATGTGTA

OCT4P1 (801) GACCATCTGCCGCTTTGAGGCTCTGCAGCTTAGCTTCAAGAACATGTGTA

OCT4A (650) AGCTGCGGCCCTTGCTGCAGAAGTGGGTGGAGGAAGCTGACAACAATGAA

OCT4P1 (851) AGCTGCGGCCCTTGCTGCAGAAGTGGGTGGAGGAAGCTGACAACAATGAA

OCT4A (700) AATCTTCAGGAGATATGCAAAGCAGAAACCCTCGTGCAGGCCCGAAAGAG

OCT4P1 (901) AATCTTCAGGAGATATGCAAAGCAGAAACCCTCATGCAGGCCCGAAAGAG

OCT4A (750) AAAGCGAACCAGTATCGAGAACCGAGTGAGAGGCAACCTGGAGAATTTGT

OCT4P1 (951) AAAGCGAACCAGTATCGAGAACCGAGTGAGAGGCAACCTGGAGAATTTGT

OCT4A (800) TCCTGCAGTGCCCGAAACCCACACTGCAGCAGATCAGCCACATCGCCCAG

OCT4P1 (1001) TCCTGCAGTGCCCGAAACCCACACTGCAG---ATCAGCCACATCGCCCAG

OCT4A (850) CAGCTTGGGCTCGAGAAGGATGTGGTCCGAGTGTGGTTCTGTAACCGGCG

OCT4P1 (1048) CAGCTTGGGCTCGAGAAGGATGTGGTCCGAGTGTGGTTCTGTAACCGGCG

OCT4A (900) CCAGAAGGGCAAGCGATCAAGCAGCGACTATGCACAACGAGAGGATTTTG

OCT4P1 (1098) CCAGAAGGGCAAGCGATCAAGCAGCGACTATGCACAACGAGAGGATTTTG

OCT4A (950) AGGCTGCTGGGTCTCCTTTCTCAGGGGGACCAGTGTCCTTTCCTCTGGCC

OCT4P1 (1148) AGGCTGCTGGGTCTCCTTTCTCAGGGGGACCAGTGTCCTTTCCTCCGGCC

OCT4A (1000) CCAGGGCCCCATTTTGGTACCCCAGGCTATGGGAGCCCTCACTTCACTGC

OCT4P1 (1198) CCAGGGCCCCATTTTGGTACCCCAGGCTATGGGAGCCCTCACTTCACTGC

OCT4A (1050) ACTGTACTCCTCGGTCCCTTTCCCTGAGGGGGAAGCCTTTCCCCCTGTCT

OCT4P1 (1248) ACTGTACTCCTCAGTCCCTTTCCCTGAGGGGGAAGTCTTTCCCCCAGTCT

OCT4A (1100) CCGTCACCACTCTGGGCTCTCCCATGCATTCAAACTGAGGTGCCTGCCCT

OCT4P1 (1298) CCGTCATCACTCTGGGCTCTCCCATGCATTCAAACTGAGGTGCCTGCCCT

OCT4A (1150) TCTAGGAATGGGGGACAGGGGGAGGGGAGGAGCTAGGGAAAGAAAACCTG

OCT4P1 (1348) TCTAGGAATGGGGAACAGGGG-AGGGGAGGAGCTAGGGAAAGAGAACCTG

OCT4A (1200) GAGTTTGTGCCAGGGTTTTTGGGATTAAGTTCTTCATTCACTAAGGAAGG

OCT4P1 (1397) GAGTTTGTGGCAGGGCTTTTGGGATTAAGTTCTTCATTCACTAAGGAAGG

OCT4A (1250) AATTGGGAACACAAAGGGTGGGGGCAGGGGAGTTTGGGGCAACTGGTTGG

OCT4P1 (1447) AATTGGGAACACTAAGGGTGGGGGCAGGGGAGTTTGGGGCAACTGGTTGG

OCT4A (1300) AGGGAAGGTGAAGTTCAATGATGCTCTTGATTTTAATCCCACATCATGTA

OCT4P1 (1497) AGGGAAGGTGAAGTTCAATGATGCTCTTGATTTTAATCCCACATCATGTA

OCT4A (1350) TCACTTTTTTCTTAAATAAAGAAGCCTGGGACACAGTAGATAGACACACT

OCT4P1 (1547) TCACTTTTTTCTTAAATAAAGAAGCCTGGGACACAGTAAA----AA----

OCT4A (1400) TAAAAAAAAAAA

OCT4P1 (1589) -AAAAAAAAAAA

**Reference**

1. Wang X, Zhao Y, Xiao Z, et al. Alternative translation of OCT4 by an internal ribosome entry site and its novel function in stress response. Stem cells*.* 2009;27:1265-1275.

2. Liedtke S, Enczmann J, Waclawczyk S, et al. Oct4 and its pseudogenes confuse stem cell research. Cell stem cell*.* 2007;1:364-366.
